# Comment on The Unbearable Lightness of Difference Between Statistical and Clinical Significance

**DOI:** 10.1097/AS9.0000000000000122

**Published:** 2022-01-03

**Authors:** Luca Bertolaccini, Oriana Ciani, Lorenzo Spaggiari

**Affiliations:** From the * Department of Thoracic Surgery, IEO, European Institute of Oncology IRCCS, Milan, Italy; † Centre for Research on Health and Social Care Management (CERGAS), SDA Bocconi School of Management, Milan, Italy; ‡ Department of Oncology and Hemato-Oncology, University of Milan, Milan, Italy.

In the latest issue of *Annals of Surgery*, Jin et al compared the efficacy of robotic-assisted (RATS) lobectomy and video-assisted (VATS) lobectomy in a single-center, open-label, parallel-arm noninferiority randomized clinical trial. They concluded that both approaches were safe and feasible, but RATS resulted in a more significant number of lymph nodes harvested.^1^ These results partially confirm the findings of a recent meta-analysis, where RATS was demonstrated as a feasible and safe technique with surgical efficacy comparable to VATS.^2^

The short-term results of the RVlob Trial are that the total number of lymph nodes dissected (11 vs 10) and the number of nodal stations examined were greater with RATS than with VATS (6 vs 5). Additionally, a more significant number of lymph nodes were dissected in N1 stations (6 vs 5) whether N2 stations remained constant (5 in both approaches).^1^ A previous meta-analysis showed that the number of dissected lymph nodes number comparing RATS with VATS does not reach a statistically significant level (>10 lymph nodes dissected).^2^ Nevertheless, it was demonstrated that examining more lymph nodes increases the likelihood of proper staging and influences the patient outcome. This information is critical for individual treatment and prognosis and for identifying those who benefit from adjuvant therapy.^3^ It was demonstrated that patients who undergo pulmonary lobectomy or pneumonectomy should have at least 12 lymph nodes examined.^4^

Nevertheless, when interpreting clinical evidence, 3 questions should be asked: How trustworthy are the findings? Are the results purely coincidental? Are the results meaningful to patient?^5^ Most journals now endorse the extension of the CONSORT 2019 statement for reporting noninferiority and equivalence randomised trials.^6^ Nonetheless, a common pitfall of this and other trial reports is the misinterpretation of *statistical significance* as *clinical significance*. The misunderstanding stems from the fact that many people associate significance with its literal meaning of significance, whereas in statistics, it has a much more restrictive connotation. In clinical practice, the clinical significance of study results is determined by their implications for current practice, with treatment effect size being one of the primary factors influencing treatment decisions. Clinical significance should be determined by the magnitude of the change, its effect on subject lives, the duration of the effects, consumer acceptability, cost-effectiveness, and ease of implementation. While established, traditionally accepted values exist for statistical significance testing, they do not exist for clinical significance evaluation. Often, the clinician’s judgment determines whether a result is clinically significant or not.^7^ Therefore, even statistically significant, the reported difference of only one lymph node dissected in the different nodal stations is clinically insignificant.

Among the secondary endpoints of the evaluation, the authors considered direct and indirect costs of the hospitalisation. According to widely used guidelines for the economic evaluation of healthcare interventions, direct costs include all costs directly related to the intervention of interest, borne inside the healthcare sector (eg, materials, equipment, personnel, and services) as well as outside the healthcare sector (eg, patients’ travel time). Indirect costs include a temporary absence from work due to illness, reduced working capacity due to illness and disability, or lost productivity due to early death. While there is an increasing interest toward measuring the indirect costs of healthcare treatments to quantify the value of innovative therapies from a broader societal perspective, what the authors report as indirect costs, does not cover this evidentiary gap. Indeed, the level of details provided on the cost analysis (eg, lack of reporting of monetary unitary values and their sources) is not sufficient to appraise the methodological quality of findings about the direct costs either.^8^

In any case, Jin et al conclude that hospitalisation and indirect costs were significantly higher in the RATS without a statistically significant difference in direct costs between minimally invasive approaches. Given the relatively anticipated high cost of the robotic approach, it was reasonable to consider the cost performance index when selecting the minimally invasive approach.^1^ RATS was estimated to cost more than VATS and open surgery, with a profit margin of 18% compared with the Italian health service reimbursement. Cost estimates for robotic surgery were 82% of lobectomy reimbursement, 69% for open surgery, and 68% for VATS. Nevertheless, the current high cost of RATS is concerning and may limit its adoption by European thoracic surgeons. However, the approach was profitable because it cost approximately 18% less than the current reimbursement for Italian health services.^9^

In conclusion, we should keep in mind that the clinical significance of study results should be determined by examining the actual treatment effect (with confidence intervals) and should not be based solely on *P* values and statistical significance. Before conducting the study, researchers should be more vigilant in using outcome measures that have a defined minimal clinically important difference or define a *minimal clinically important difference*. If the outcome of interest does not have a defined minimum clinically important difference, then researchers should be wary of simply interpreting results as *significant* based on a *P* value without considering the clinical impact of the findings.^10^
